# COVID-19 infection, hospitalisation and mortality rates in people with severe mental illness: findings from two UK cohort studies

**DOI:** 10.1192/j.eurpsy.2022.234

**Published:** 2022-09-01

**Authors:** L. Hassan, J. Firth

**Affiliations:** University of Manchester, Division Of Psychology & Mental Health, Manchester, United Kingdom

**Keywords:** UK, Covid-19, SMI, Electronic Health Records

## Abstract

**Introduction:**

Recent systematic reviews have highlighted that people with Severe Mental Illness (SMI) have higher risks of infection, hospitalisation and death from COVID-19, although the full extent of these disparities are not yet established.

**Objectives:**

Utilising electronic health records, we investigated COVID-19 related infection, hospitalisation and mortality among people with schizophrenia/psychosis, bipolar disorder (BD) and/or major depressive disorder (MDD) in two large UK samples: the UK Biobank (UKB) cohort study and GP-registered patients in Greater Manchester (GM).

**Methods:**

We sampled 447,296 adults with and without SMI from UKB (inc. schizophrenia/psychosis=1,925, BD=1,483 and MDD=41,448, non-SMI=402,440) and 1,152,831 adults from GM (inc. schizophrenia/psychosis =46,859, BD=3,461, recurrent MDD=134,661, non-SMI = 922,264). Primary care, hospital and death records were linked to identify COVID-19 related outcomes. Logistic regression models were used to estimate unadjusted and adjusted Odds Ratios (ORs) to compare differences in COVID-19 outcomes by diagnosis, controlling for sociodemographic factors and comorbidities.

**Results:**

We will report the findings of unadjusted and adjusted analyses, comparing ORs for people with and without SMI, by diagnosis. Findings will be compared between the two datasets, with attention to the demographic and clinical profiles of each sample. We will consider the role of demographic characteristics and comorbidities in attenuating outcomes.

**Conclusions:**

Emerging evidence suggests that people with SMI have higher risks of COVID-19 infection, hospitalisation and mortality. Based on two large datasets utilising EHRs, we present findings from the UK on COVID-19 outcomes among people with SMI, a country that has been severely affected by COVID-19.

**Disclosure:**

No significant relationships.

